# Conservation Status of North American Birds in the Face of Future Climate Change

**DOI:** 10.1371/journal.pone.0135350

**Published:** 2015-09-02

**Authors:** Gary M. Langham, Justin G. Schuetz, Trisha Distler, Candan U. Soykan, Chad Wilsey

**Affiliations:** 1 National Audubon Society, Washington, DC, United States of America; 2 National Audubon Society, San Francisco, California, United States of America; Cary Institute of Ecosystem Studies, UNITED STATES

## Abstract

Human-induced climate change is increasingly recognized as a fundamental driver of biological processes and patterns. Historic climate change is known to have caused shifts in the geographic ranges of many taxa and future climate change is expected to result in even greater redistributions of species. As a result, predicting the impact of climate change on future patterns of biodiversity will greatly aid conservation planning. Using the North American Breeding Bird Survey and Audubon Christmas Bird Count, two of the most comprehensive continental datasets of vertebrates in the world, and correlative distribution modeling, we assessed geographic range shifts for 588 North American bird species during both the breeding and non-breeding seasons under a range of future emission scenarios (SRES A2, A1B, B2) through the end of the century. Here we show that 314 species (53%) are projected to lose more than half of their current geographic range across three scenarios of climate change through the end of the century. For 126 species, loss occurs without concomitant range expansion; whereas for 188 species, loss is coupled with potential to colonize new replacement range. We found no strong associations between projected climate sensitivities and existing conservation prioritizations. Moreover, species responses were not clearly associated with habitat affinities, migration strategies, or climate change scenarios. Our results demonstrate the need to include climate sensitivity into current conservation planning and to develop adaptive management strategies that accommodate shrinking and shifting geographic ranges. The persistence of many North American birds will depend on their ability to colonize climatically suitable areas outside of current ranges and management actions that target climate adaptation.

## Introduction

Climate change presents a great challenge: balancing the conservation needs of species today while preparing for largely unknown responses in the future [1–7]. Uncertainty around the biological responses of species to climate change makes informed management actions difficult; however, adaptive management frameworks can be developed once species-level assessments become available (e.g. [5,8]). As one of the best-studied groups in the world, birds offer an opportunity to model climate change responses for a taxon as a whole, at large geographic scales, and with sufficient spatial resolution to be meaningful for on-the-ground conservation. Furthermore, since birds are so widespread and cover so many habitats, birds are a reasonable proxy for the overall implications of climate change for all wildlife in North America. Previous climate studies of North American birds have focused on responses dealing with few species or limited geographies [9–12], or global studies done at coarse scales [2,7,13,14]. Overcoming these constraints requires large amounts of data, including both numerous species and broad spatiotemporal coverage.

While local and regional species’ distributions tend to be influenced primarily by biotic interactions and habitat and/or resource availability, respectively, climate influences broad-scale distributions putatively through interactions with the physiological limits of the organism [7,15–18]. Modeling changes in geographic range, therefore, provides a concise and intuitive measure of potential climate sensitivity and accompanying level of conservation risk associated with climate change [16,19–21].

Lists of priority species [22–23] are valuable conservation tools useful for defining management priorities and goals, but typically do not include assessments of conservation risk due to future climate change. Climate sensitivity can be included in future species vulnerability assessments to help prioritize conservation investments and to provide testable predictions for future monitoring of at-risk species [5,18]. Here we model the geographic ranges of 588 North American bird species across an array of future climate-emissions scenarios in both breeding and non-breeding seasons, and we use the results to assess the climate sensitivity of each species to inform conservation planning.

## Materials and Methods

### Bird Data

Presence/absence data were obtained from two sources: the North American Breeding Bird Survey (BBS) [21] and the Audubon Christmas Bird Count (CBC) [22]. These surveys are remarkable in the breadth of spatial coverage and the use of consistent sampling methods. The BBS monitors bird populations in the United States and Canada between early May and mid-July. Survey routes are 39.4 km long with 50 stops. At each stop, participants conduct a 3-min point count and record birds seen or heard. We pooled observations of each species over the first 30 stops (~24 km) for each BBS route and distilled counts into presence/absence data. This allowed us to maintain a reasonable match to the resolution of the climate grids that we used for projections (10 x 10 km) and to balance the scales of BBS and CBC samples used in our models. The CBC began in 1900 to document early winter bird assemblages in North America and has a growing presence throughout the Western Hemisphere. CBC surveys are conducted within 24.1 km-diameter circles for one 24 h period during a two-week interval centered on December 25. For this study, all circles that fell within the boundaries of the United States and Canada were included. For every circle and count year, we distilled raw count data into presence/absence information for each species. Since all information was collected from existing datasets and no animals were handled, no specific permission was required for this project.

### Climate Data

Historical climate data were obtained from the Canadian Forest Service (CFS) and linked to historical bird observations using a point extraction tool that provided spatially continuous estimates of climate across the landscape [23, 24]. By using the mid-point of each CBC circle and the start-point of each BBS route as extraction points we ensured the distance between climate estimates and bird observations never exceeded 24 km. This approach seemed reasonable given the structure of the data sets, the continental scale of the study, inherent uncertainty in historical climate estimates, and the lack of systematic bias in the association of climate estimates and bird observations (even when small spatial mismatches may have occurred). We matched bird data and climate data on an annual basis, assuming that climate variables from the year leading up to each survey would best inform our understanding of occurrence data. We used 17 bioclimatic variables to characterize conditions in each year.

Bioclimatic variables are thought to represent biologically meaningful combinations of monthly climate variables because they aggregate climate information in ways known to drive biological processes [25]. Bioclimatic variables can be highly correlated, however, which raises concerns about the ability of modeling algorithms to identify causal relationships in data sets and to generate accurate predictions [26]. A variety of approaches to clustering or removing predictors can be employed in an effort to reduce collinearity but none of them guarantee better inference or prediction [26]. Ultimately, iterative assessment of correlated variables through observation and experiment is needed. We chose not to remove variables from our data set because we had little or no *a priori* justification for choosing one correlated variable over another. In addition, our approach to matching bird data and climate data on an annual basis (described below) allowed correlations to vary across time and space, thus reducing the degree of collinearity.

### Species Distribution Models

Species distribution models were developed using boosted regression trees (BRTs) [27]. BRT models aim to improve predictive performance of any single model by incorporating tree-based methods that iteratively fit nonlinear relationships and handle interactions between predictors automatically [28]. We built separate distribution models for breeding and non-breeding seasons using BBS and CBC data, respectively. Our analyses of BBS and CBC data were similar in approach with small adjustments to account for differences in data sets and survey protocols. In addition to bioclimatic variables, for BBS analyses we used ordinal date (day of year) to account for variation in timing of surveys across the summer season. We used 25081 records collected along 3718 routes from 2000–2009 to train our models and 41959 records collected from 1980–1999 to test the predictive performance of our models. This approach allowed us to take advantage of increased geographic sampling in recent years to build models and availability of abundant historical data for assessing the predictive ability of our models outside the current time period and climate space. We had sufficient data to construct models for 475 North American species during the breeding season (see Supporting Information). For CBC analyses, we included the number of survey hours invested in each CBC circle as a predictor to account for uneven observer effort across circles, in addition to bioclimatic variables. The number of participating individuals and the duration of counts vary among CBC circles and through time, thus the number of party-hours has often been used as a covariate to account for this variation in analyses based on CBC data (e.g., [29]). We used 19272 records collected at 2278 circles from 2000–2009 to train our models and 30630 records collected from 1980–1999 to assess the predictive ability of our models. We had sufficient data to construct models for 503 North American species during the non-breeding season (see Supporting Information).

We built models using the following parameters: (i) learning rate = 0.01, (ii) tree complexity = 5, (iii) family = Bernoulli. These settings resulted in models built with an average of 3100 and 2800 trees for winter and summer species, respectively, well beyond the suggested minimum of 1000 [28]. All models were built using the gbm package [27] in R and cross-validation was performed using 10 folds.

### Spatial Projections and Range Delineation

We generated current species distributions by projecting modeled relationships across gridded annual bioclimatic data averaged from the same period used to construct the models. A spatial layer containing the median survey day was included as a constant in projections of BBS models and a layer containing the median number of survey hours was included as a constant in projections of CBC models. To characterize future climates and establish a spatial context for projections, we added future climate anomaly grids to baseline climate data in several processing steps. First, we obtained spatially downscaled (5-min resolution) climate grids for 13 combinations of general circulation models (GCMs) and emissions scenarios [30] summarized across each of three future time periods (2010–2039, 2040–2069, 2070–2099; hereafter 2020, 2050, 2080). We then subtracted contemporary WorldClim grids [31] for monthly minimum temperature, maximum temperature, and precipitation from the future grids to isolate predicted climate anomalies from baseline values. This resulted in a change value for each of the three variables between current and future for each month. Finally, we added these monthly anomaly grids to CFS mean climate grids for the base period (1971–2000) for each variable in each month, ensuring correspondence between the historical climate used to build the models and simulated future climate [32]. We then transformed projected temperature and precipitation data into bioclimatic variables using DIVA software and the raster package [33] in the statistical software R.

Species distribution models were projected into each of 39 future climate surfaces (i.e., 13 combinations of emissions scenarios and GCMs in each of 3 future time periods). Because we were fundamentally interested in understanding responses of species to climate change across scenarios and time, we ensembled projections for multiple GCMs within each scenario and time period (consensus forecasting *sensu* [34]). This process resulted in 9 future prediction grids for each species, one for each emissions scenario (SRES A2, A1B, B2) in each time period (2020, 2050, 2080) that described climatic suitability of the United States and Canada on a continuous scale from 0 (unsuitable) to 1 (highly suitable). When estimating range sizes, we delineated boundaries of species ranges using a threshold value based on the maximum Kappa statistic [35]. The Kappa statistic measures the proportion of correctly predicted sites after the probability of chance agreement has been removed [36]. We chose the maximum Kappa statistic because it provided us a conservative estimate of ranges compared to alternative thresholds, particularly for rare species. We used raster [33] and dismo packages [37] in R for all projections and raster manipulations.

### Climate Sensitivity

We assigned each species to a climate sensitivity status based on overall changes in climatic suitability: *Climate endangered*, *climate threatened*, or *climate stable*. We defined *climate endangered* as projected loss of more than 50% of current range by 2050 across all scenarios, with no net gain from potential range expansion. *Climate threatened* was defined as projected loss of more than 50% of current range by 2080 across all scenarios, with net gain from potential range expansion possible. *Climate stable* was defined as projected loss of less than 50% of current range across scenarios. *Climate stable* included 46 species, defined as introduced (*n* = 14) or native species with ranges that occur only marginally within our study area (*n* = 32).

### Conservation Priority Ranks

We assessed how well our climate sensitivity rankings matched three existing conservation priority assessments: Partners in Flight (PIF) maximum threat scores [38], IUCN Red List (IUCN) [39], U.S. federal status and Birds of Conservation Concern (FED/BCC) [40]. Federal status and Birds of Conservation Concern assessments were combined because the BCC list was developed to complement the federal list. For this composite category each species was assigned to one of three groups: FED, BCC, or neither. Measuring the relationship between Audubon climate sensitivity status and existing conservation priority assessments required that we assign numerical ranks to species based on their conservation status. PIF scores were already numerical, but Audubon climate sensitivities, FED/BCC, and IUCN categories had to be assigned ranks. We converted Audubon climate risk status to rankings, with the highest threat being assigned the lowest rank and the *climate stable* group being assigned the highest rank (marginal and introduced species were excluded from this analysis as many of them are not classified according to one or more of the assessments). For FED/BCC, species were assigned a rank of 1 if they were federally listed, 2 if they were of conservation concern, and 3 if neither label applied. For the IUCN categories, species were assigned a rank of 1 if they were considered endangered, 2 if threatened, and 3 if considered to be of least concern. We then used Kendall rank correlation analysis to examine the relationships between climate sensitivity and existing conservation priority assessments (one correlation for each comparison—Audubon vs. PIF, Audubon vs. IUCN, and Audubon vs. FED/BCC).

### Geographic Shifts in Species Distributions

We characterized changes in species distributions with respect to latitude, elevation, and distance from the coast to evaluate whether distributional responses to climate change might generalize across species. Simple hypotheses that focus on the role of cold temperature in limiting distributions predict geographic shifts northward, upslope, and inland as overall warming trends continue throughout the century (e.g., [14]). To characterize potential future shifts, we summarized latitude, elevation, and distance to coast values across current and future species distribution grids. Values in each grid cell were weighted by the proportion of each species distribution in the corresponding cell and averaged across all cells, resulting in centroid estimates of latitude, elevation, and distance to coast for each species in current and future time periods.

### Covariates and Dissimilarity

Variance components analysis is a way to assess the amount of variation in a dependent variable that is associated with one or more predictor variables [41]. The central output is a variance components table that shows the proportion of variance attributable to each variable (note: the predictor variables have to be treated as random effects for this analysis). We used the lmer function, which is part of the lme4 package in R [42] to run random-effects ANOVAs to ascertain the percent of variation in current geographic range that is explained by four covariates relevant to bird conservation: species, migratory strategy [43], climate change scenario (SRES A2, A1B, B2), and habitat affinity (IUCN). This was done separately for both the breeding and non-breeding seasons. We also ran the same analyses, using relative change in range size as the response variable. Finally, we repeated all four variance components analyses using Audubon’s habitat classifications to see if choice of habitat assignment scheme affected the results.

To estimate changes in community composition between the present and future, we used the vegdist function, part of the vegan package in R [44], to calculate binary Bray-Curtis dissimilarity (equivalent to 1—Sørensen similarity) for each grid cell. Dissimilarity was calculated for each season, comparing the present and 2080 (SRES A2). Dissimilarity results were then mapped to provide a community-level complement to estimates of species gains and losses derived from changes in individual species ranges.

## Results

**Fig 1:**
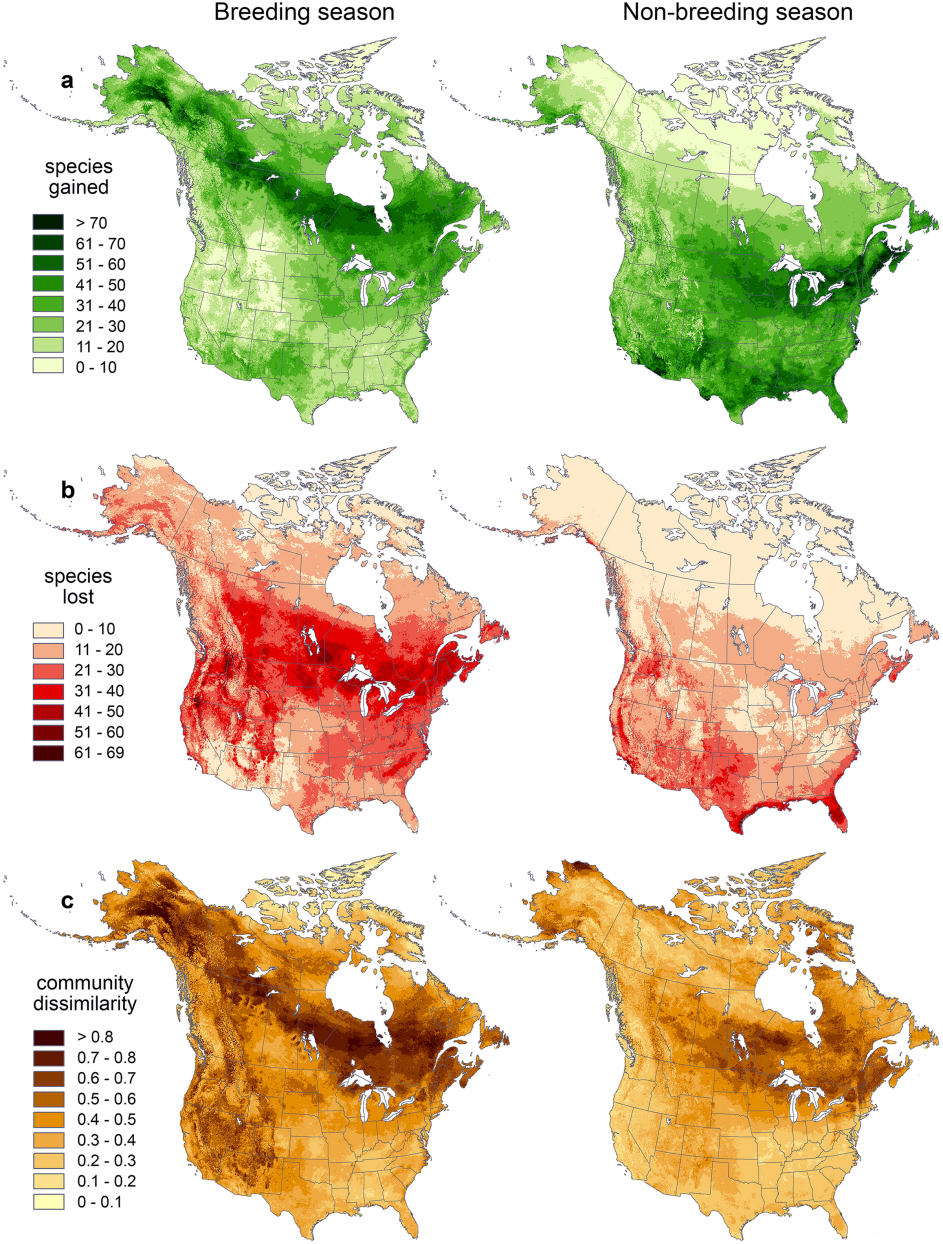
Impacts of future climate change on geographic ranges of North American bird species (*n* = 588) by 2080 during the breeding (top row) and non-breeding season (bottom row) under SRES A2 scenario. Changes in total number of species by 2080 due to shifting ranges relative to year 2000 baseline showing potential **(a)** species gain or **(b)** loss. **(c)** Bray-Curtis dissimilarity showing turnover in species composition of local communities.

Our study suggests that by 2080, under a high-emissions scenario (SRES A2), breeding and non-breeding ranges of North American birds are projected to change dramatically in size and location. These changes will produce distinct patterns of species gains and losses across the continent (Fig 1). During the breeding season, bird communities may gain as many as 80 species, centered on the far northern Canadian provinces, especially boreal forests, and central Alaska, and lose as many as 69 species (Fig 1a and 1b). Peak areas of loss include much of the area along the U.S.-Canadian border where Eastern deciduous forests, prairie potholes, as well as the high elevations of the Rockies and Sierra ranges occur. During the non-breeding season, bird communities may gain as many as 92 species, mainly in the border regions with Canada and Mexico, and may lose as many as 65 species (Fig 1a and 1b). Peak areas of loss in the non-breeding season are centered on central and southern California, the Gulf region, and southern coastal states (Fig 1a). Future mid- and low-emissions scenarios (SRES A1B and B2, respectively) show patterns of species gain and loss through time that are broadly similar, in both magnitude and location, to projections from the A2 scenario (Table 1; see also [30]). Details on species gains and losses by state or province, Landscape Conservation Cooperative, or Bird Conservation Region are presented in S1–S4 Appendices.

**Table 1:** Number of modeled North American bird species in each climate sensitivity category by season (summer and winter), scenario (SRES A2, A1B, B2), or either season across all scenarios.

		Climate Sensitivity			
Season	SRES Scenario	Loss by 2050 ^a^	Loss by 2080 ^b^	<50% loss ^c^	Other ^d^
**summer (*n* = 468)**	A2	101	182	159	26
	A1B	101	145	196	26
	B2	123	135	184	26
	All three	85	148	209	26
**winter (*n* = 503)**	A2	71	100	289	43
	A1B	71	93	296	43
	B2	92	76	292	43
	All three	59	90	311	43
**summer or winter (*n* = 588)**	All three	126	188	228	46

^a^ >50% loss current range by 2050 and potential gain < loss.

^b^ >50% loss of current range by 2080.

^c^ <50% loss any year.

^d^ introduced or marginal species.

Combining projected range gains and losses suggests that future bird communities will differ markedly from current communities, particularly during the breeding season (Fig 1c). For the SRES A2, 2080 climate change scenario, mean dissimilarity between the present and future was 0.470 for the breeding season and 0.374 for the non-breeding season. In the breeding season, dissimilarity was greatest in the boreal forests and western United States. In the non-breeding season, dissimilarity was greatest around the Great Lakes and in northern Alaska.

**Fig 2:**
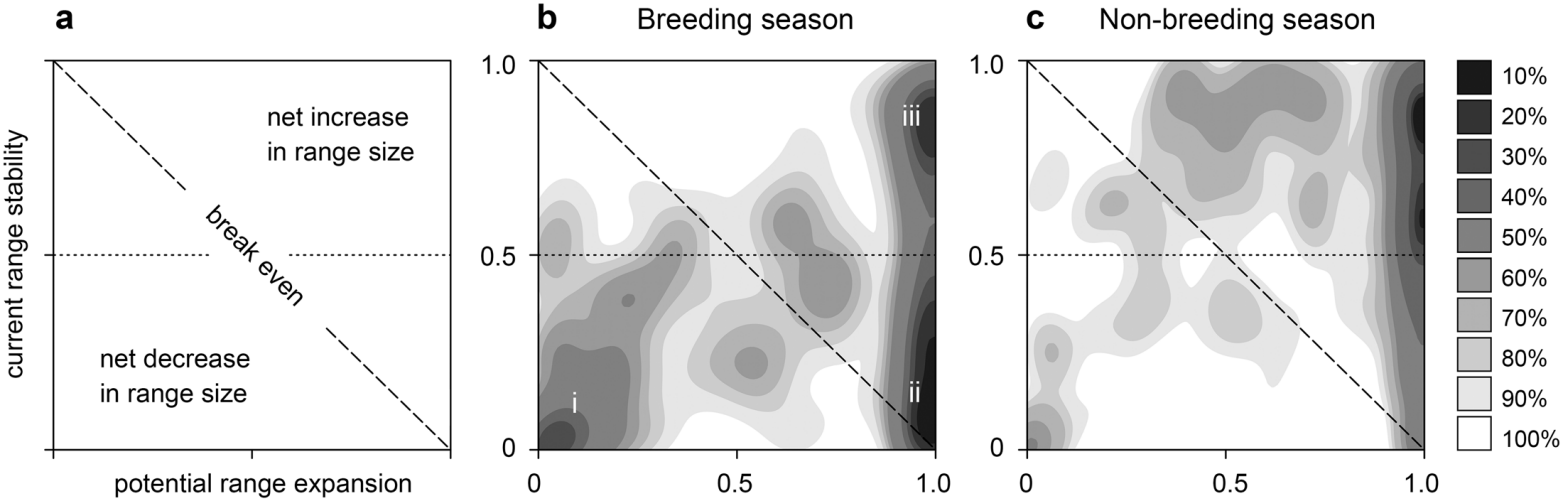
Relationship between current range stability (year 2000 range remaining in 2080) and potential range expansion (proportional to current range size) under SRES A2 scenario by year 2080. **(a)** Two-dimensional density plot (10% contours) of North American bird species showing current range stability versus potential range expansion for **(b)** breeding (*n* = 475) with three distinct groups: (i) current geographic range decreases with little to no expansion potential; (ii) current geographic range shifts to other parts of North America; and, (iii) current geographic range remains stable and, in some cases, expands, and **(c)** non-breeding seasons (*n* = 503). Species with expansion values >1.0 were truncated to 1.0 to appear on plots.

**Fig 3:**
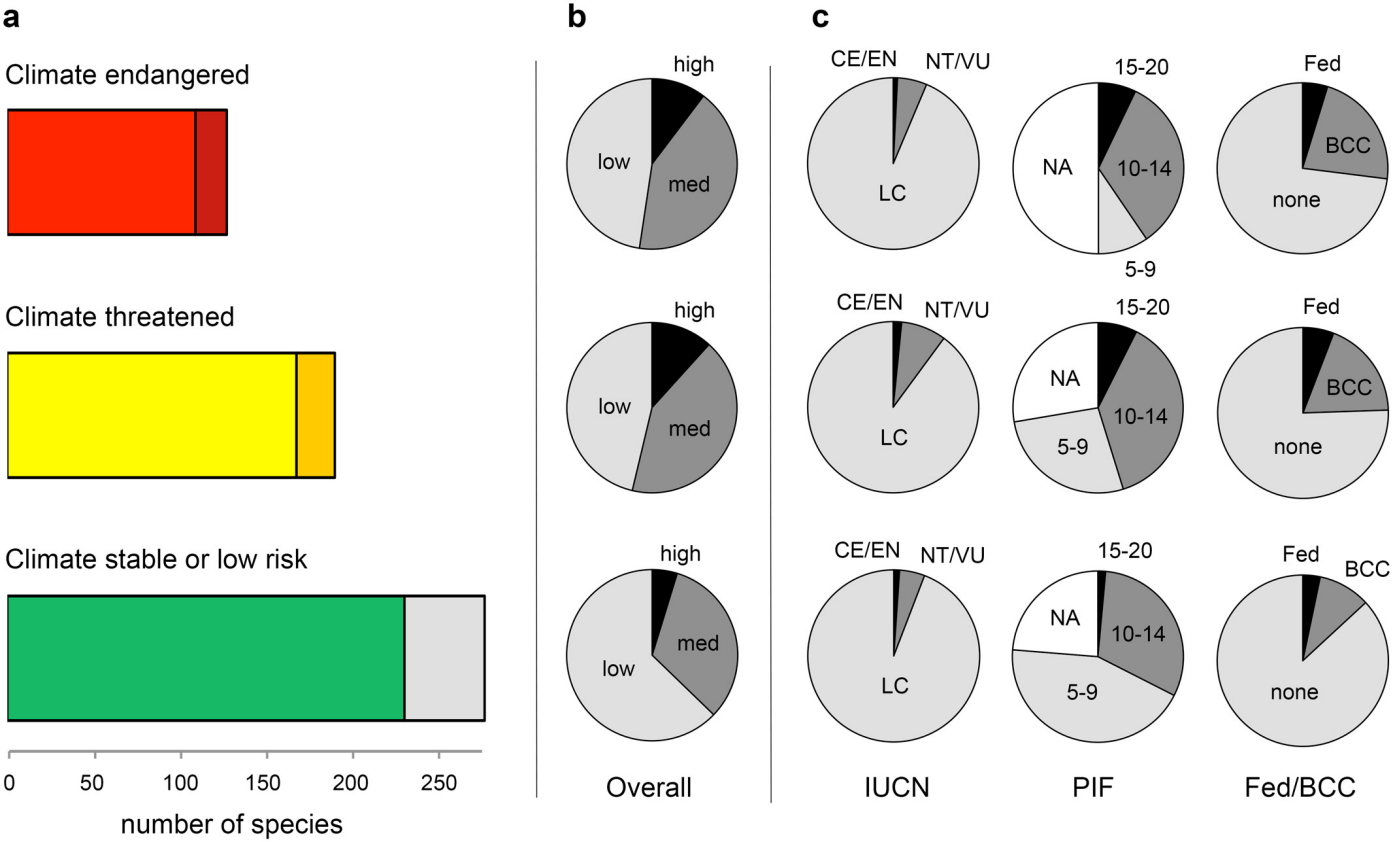
Climate risk from future climate change across North American bird species (*n* = 588) in relation to existing conservation assessments. **(a)** Audubon climate risk categories: *Climate endangered* in one season (red, *n* = 108) or both (dark red, *n* = 18); *climate threatened* in one season (yellow, *n* = 166) or both (dark yellow, *n* = 22); and *climate stable* (green, *n* = 228), or *other* covering marginally occurring or introduced species (gray, *n* = 46). **(b)** Highest conservation risk status. **(c)** International Union for Conservation of Nature (IUCN) Red List (black = critically endangered and endangered, dark gray = near threatened and vulnerable, and gray = least concern), Partners in Flight (PIF) threat scores (black = 15–20, dark gray = 10–14, gray = 5–9, and white = unranked), and U.S. federal status (Fed = black) and Birds of Conservation Concern (BCC = gray).

Our models suggest that North American birds will fare far worse during the breeding season than the non-breeding season (Fig 2; Table 1). In the breeding season, species cluster into three distinct groups (Fig 2b): (i) current geographic range decreases with little to no expansion potential; (ii) current geographic range shifts to other parts of North America; and, (iii) current geographic range remains stable and, in some cases, expands. Based on loss of current climatic range and potential gain of new climate range (see Methods), we classified 126 species (21.4%) as *climate endangered*, an additional 188 species (32%) as *climate threatened*, and 274 (46.6%) as *climate stable* (*n* = 228) or *other* (introduced and marginal spp.) (*n* = 46) (Fig 3a; Table 1).

Relatively few bird species in our *climate endangered* or *climate threatened* categories are of current conservation priority (Fig 3b and 3c; Table 1, S7 Appendix). There was no association between climate sensitivity and IUCN Red List ranking (Kendall rank correlation = 0.031, *n* = 542, *p*-value = 0.437; Fig 3c). Species that are currently considered more threatened according to FED/BCC and PIF ranking were significantly more likely to be in a higher sensitivity category due to climate change but the relationship was not strong for either priority assessment (FED/BCC: Kendall rank correlation = 0.129, *n* = 542, *p*-value = 0.001; PIF: Kendall rank correlation = 0.246, *n* = 370, *p*-value < 0.001; Fig 3c).

**Fig 4:**
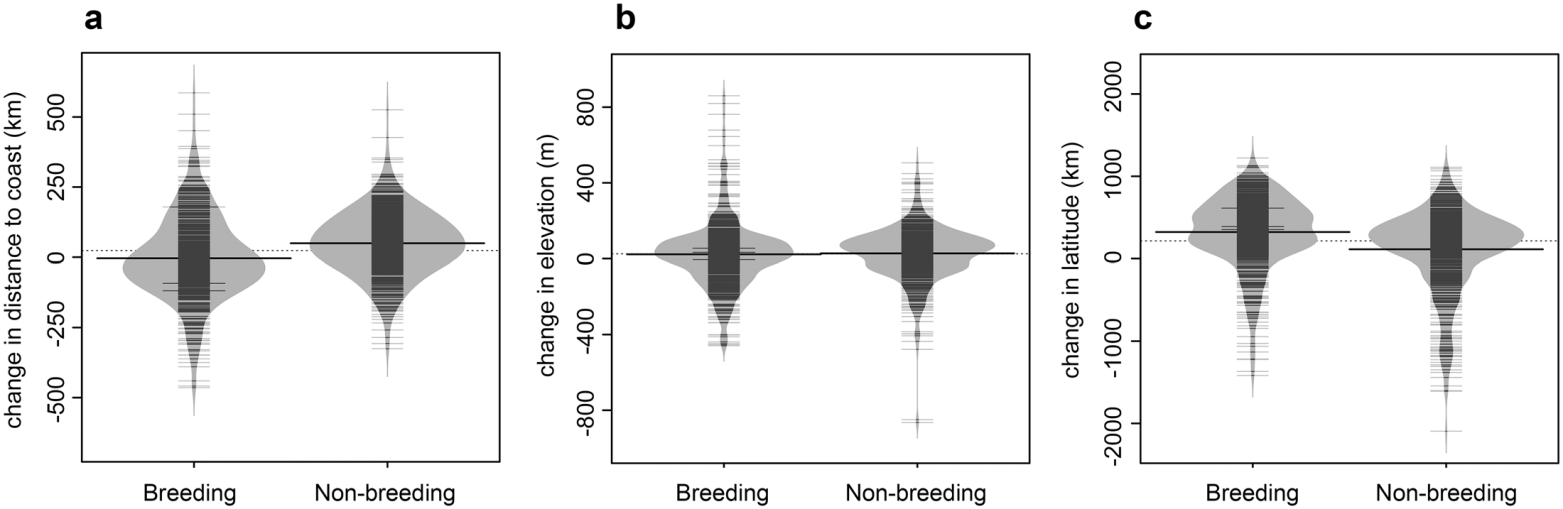
Projected geographic shifts in breeding and non-breeding distributions from 2000 to 2080 under a high-emissions (SRES A2) scenario. Change in species distributions were calculated using weighted mean values for **(a)** distance from the coast **(b)** elevation and **(c)** latitude. Dotted lines indicate the mean across seasons. Black lines indicate medians within each season.

Shifts in the geographic locations of species distributions were heterogeneous (Fig 4). While distributions were estimated to shift northward an average of 322 km during the breeding season and 116 km during non-breeding season (SRES A2, 2080), forecasts varied considerably, with 16% of breeding taxa and 27% of non-breeding taxa projected to shift southward.

Projections suggest that climate change will tend to push species toward higher elevations through the end of the century (SRES A2, 2080) (mean change in elevation across all species: breeding season: 22 m upslope; non-breeding: 29 m upslope). Many species are projected to move downslope, however (breeding season: 41% of taxa; non-breeding season: 36% of taxa). Variation in elevational responses across species underscores the importance of looking at individual species when estimating climate disruption. Over the same time interval and scenario, species distributions are expected to shift inland an average of 51 km during the non-breeding season and 4 km coastward during the breeding season (Fig 4).

**Fig 5:**
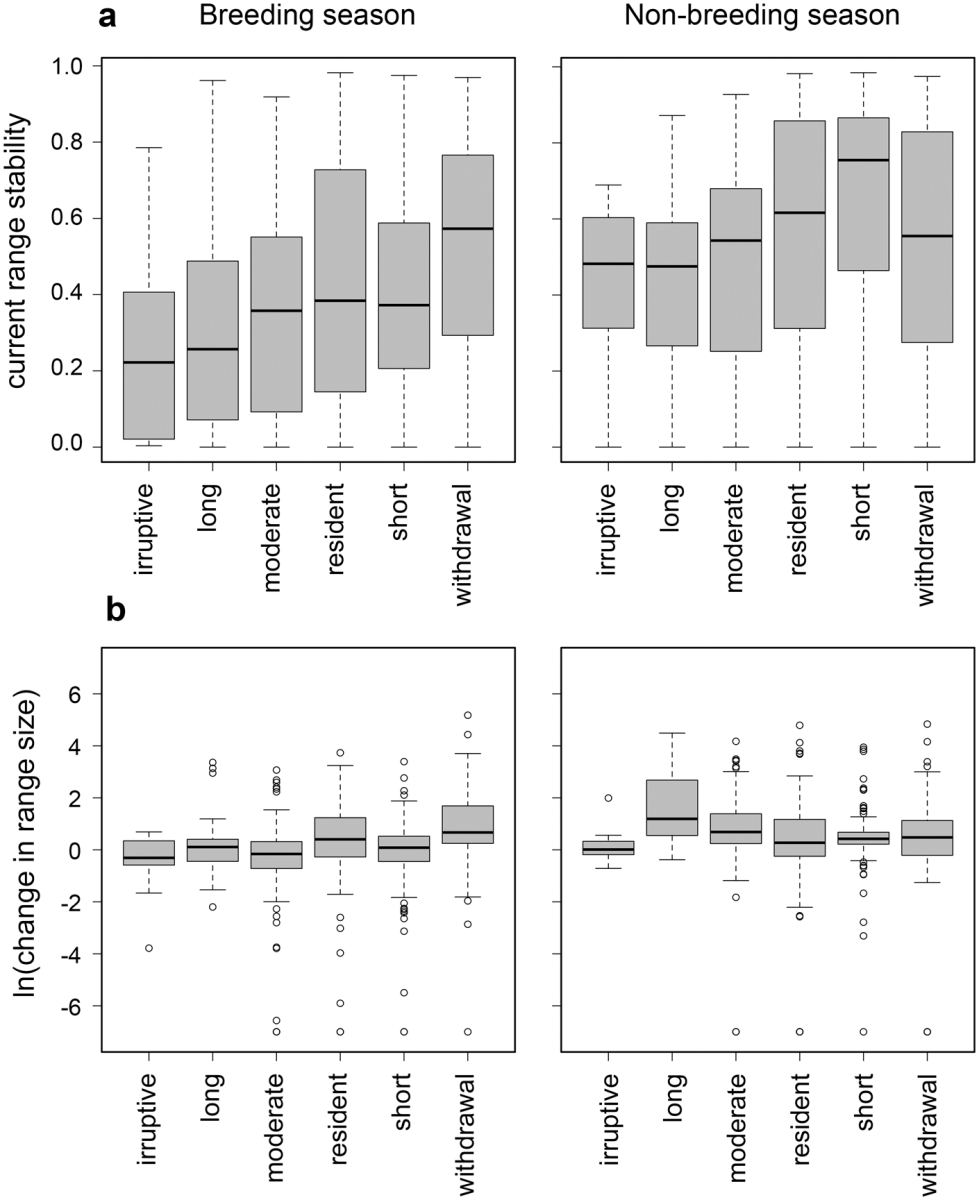
Stability and change in geographic range size in the breeding and non-breeding season in relation to migratory groups. **(a)** Change in current geographic range size (year 2000) by 2080 (SRES A2) grouped by migratory behavior (S5 and S6 Appendices). **(b)** Proportional change in range size by 2080 (SRES A2) grouped by migratory behavior (S5 and S6 Appendices).

The vast majority of variation in projected responses to climate change was due to individual species effects (~69–94%; S5 Appendix). Species’ habitat affinities based on the IUCN classification accounted for essentially none of the variation in current range stability or relative change in range size in either season (S5 and S6 Appendices). Habitat affinities based on the Audubon classification were higher (e.g., 2–13%), but still small relative to individual species effects (S5 and S6 Appendices). Migratory behavior explained a small amount of variation in relative change in range sizes during the breeding season (~2–7% variation) and in stability of current ranges during the non-breeding season (~1–7% variation; Fig 5; S5 and S6 Appendices). Somewhat surprisingly, there was little difference (<1% variation) in projected gains or losses among climate scenarios (S5 Appendix).

All models that performed relatively well for the dataset (<2 standard deviations from the mean) were included in the study. We conducted this evaluation based on AUC and deviance explained and further excluded models based on their disagreement with known species distributions evaluated by expert review. Test data were typically from the same locations as the training data, but they were sampled in different decades and modeled with different climates. To explore whether this biased performance metrics, we recalculated performance metrics using only the 558 and 580 (BBS and CBC) locations surveyed between 1980 and 1999 that were not surveyed in the 2000–2009 training dataset. This provided both a temporally and spatially independent test of model performance. Winter model performance improved with the spatially independent dataset. Performance of summer models improved according to AUC and deviance explained but declined according to True Positive Rate (TPR). Overall, our original model performance metrics were robust to the lack of spatial independence in the test dataset (S8 and S9 Appendices).

## Discussion

This study presents the first detailed analysis of the impacts of climate change on 588 species of birds in the breeding and non-breeding season across the continental United States and Canada. Grouping species’ range changes according to our climate sensitivity criteria results in over half (314 of 588) being classified as climate endangered or threatened in this century across multiple future climate projections, including even a moderate emissions scenario (SRES B2). Although these species will lose over half of their current climatically suitable ranges in this century, most are not on any conservation priority list. To persist through the end century, our results suggest these at-risk species will need additional monitoring beyond CBC and BBS to track disruption from climate change and management to prioritize and protect current and future ranges.

### Climate Sensitivity of North American Birds

*Climate-endangered* species (21.4%) are projected to lose more than half of current climatic range by 2050 without the potential to make up losses with gains in new areas. The climate-endangered group clearly faces the highest risk of extinction or extirpation from climate change. Current management prescriptions often focus on site-level resilience or helping species track their shifting climate niche [11,12,45]. Neither action seems likely to help species with shrinking geographic ranges. For example, high elevation and high latitude species groups are often thought to face the problem of shrinking geographic ranges without expansion potential [3,5,9,46]. Conservation of these shrinking range species requires the identification of areas within their current range that will remain climatically suitable into the future. In addition, since many *climate-endangered* birds are not currently on any conservation priority list, there is no formal monitoring or legal protection for these most at risk species. For example, Baird’s Sparrow (*Ammodramus bairdii*) is projected to lose more than 95% of current range, yet is listed as a species of Least Concern on the IUCN Red List. Although listed as a priority species in some states (e.g., Wyoming; [47]), it is not in others (e.g. Colorado; [48]). We suggest that the 126 species in this category be considered by conservation entities for immediate monitoring beyond existing programs such as BBS and CBC.

*Climate-threatened* species (32%) are projected to lose more than half of current climatic range by 2080, but show potential to make up losses in new areas. With climatic ranges shifting through geographic space, these species will depend on natural or assisted movement to new locations. Shifting ranges are a familiar concept for managers. Our assessment identifies which species are potential shifters (i.e., *climate threatened*) and, when coupled with 10 × 10 km species-level maps (S1 and S7 Appendices), where this shifting is most likely to occur. This directs conservation attention to places most suitable under future climate conditions. However, even if species are able to perfectly track their climatic niches, many may suffer due to shifts in other ecological realities (e.g., competition, predation) relating to changes in the overall community. Note that it is also possible for shifting species to benefit from new interactions (e.g., exploiting new food sources or novel interactions leading to a competitive advantage for nesting).

*Climate stable* species (46.6%) are defined as species projected to maintain more than half of current climatic range across all scenarios and timeframes. Many stable species also have the potential for dramatic expansion (Fig 2biii and 2c) in addition to core range stability. This category is of lower conservation concern when thinking about climatic stability of geographic range. However, a climatically stable species could still be at risk from traditional conservation threats (e.g., habitat loss), and indirect effects of climate change that we were unable to model. For example, a rocky intertidal species could be climate stable but still experience significant threats from sea level rise, shifts in prey base, or competitors/predators shifting under climate disruption. In addition, our assessment may underestimate risks for Neotropical migrants because we were unable to model their responses to climate change on wintering grounds in Central and South America. Thus, a Neotropical migrant categorized as *climate stable* based on the breeding season in North America may be more vulnerable than predicted if its range in the non-breeding season is strongly impacted. Future work needs to be done to better understand climate change impacts across the life cycle, and impacts on demographic processes not covered in this study [49].

### Climate Sensitivity and Conservation Prioritizations

Our analyses show that current conservation prioritizations do not correspond closely with our estimates of climate sensitivity (Fig 3b and 3c). Instead current prioritizations seem to be evenly distributed across climate sensitivity categories (e.g., 60 of the 126 *climate-endangered* birds are not on any of the current prioritizations). A similar pattern has been observed elsewhere when climate sensitivity was assessed by expert opinion [50]. Thus, the causes of conservation threat today are seemingly independent of future climate sensitivity. However, any species that is already of conservation concern, and is climate endangered, is more at risk than a species of least conservation concern. Although decisions on how to balance projections of the future with present conservation status will vary depending on context, conservation assessments should incorporate information on future climate sensitivity into a formal decision-making framework centered on adaptive management. For *climate-endangered* species already of concern, conservation efforts should focus on protecting areas that are already occupied and are expected to remain suitable under future scenarios. These climate strongholds are paramount for the long-term protection of the species. For *climate-threatened* birds already of concern, this study suggests where these species will be able to move in the future, aiding planning efforts for assisted migration. *Climate stable* species will need the least amount of management, but should still be monitored to ensure that indirect consequences of climate change do not result in rapid range contractions or population declines.

### Idiosyncratic Responses to Climate Change

The relatively small amount of variation in future range explained by climate change scenario, habitat affinity, and migratory strategy should not be misinterpreted as signaling that these factors are unimportant. The minor differences in response across climate change scenarios for most species probably reflects the broadly similar trajectories of SRES scenarios until late in the century [32]. These results underscore the idiosyncratic nature of projected bird responses to future climate change. Similarly, elevational shifts were very modest when averaged across species in both seasons, but showed considerable variation, consistent with the diversity of responses recently documented (e.g., [46]). Geographic responses to climate change varied widely among species and across seasons with greater northward shifts during the breeding season and greater shifts inland during the non-breeding season. Finally, species gains (Fig 1b and 1c) along the border with Mexico would very likely increase if more Neotropical species were included in the analysis.

### Survey Effort and Occupancy Modeling

Inclusion of survey effort and survey timing data for CBC and BBS models, respectively, allowed us to generate effort-adjusted and time-adjusted predictions of occurrence analogous to those produced in other modeling efforts [51]. Occupancy modeling [52] is an alternative approach sometimes used in species distribution models that might have allowed us to characterize occurrence probability as a function of bioclimatic variables while accounting for detection probability as a function of survey effort or timing. Neither CBC nor BBS survey data are well suited for occupancy modeling, however, particularly with respect to meeting assumptions about population closure across time and space. In addition, it remains unclear how well detection and occurrence functions can be distinguished with current methods and some authors suggest the risks associated with more complex occupancy models outweigh the benefits [53].

## Conclusion

Although the level of future emissions has a statistically significant effect on projected changes in species range sizes, even the most optimistic scenario generates major shifts in species ranges. Thus, while recognizing the importance of mitigation efforts to reduce greenhouse gas emissions, we must also promote adaptation efforts to ameliorate the effects of climate change on bird populations. The long-term persistence of many bird species threatened by climate change will depend on their ability to colonize new areas made suitable due to climate change and the conservation management of areas that are presently suitable and will remain so into the future. Monitoring of species’ responses and projected climate risk, coupled with adaptive management of their populations, is likely the most effective way to deal the inherent uncertainty of climate responses. This approach creates a rigorous science framework in which continuous assessment can be linked to conservation investment. We suggest that agencies and NGOs adopt such an approach to dealing with climate change in relation to birds (and other organisms), considering species’ responses to both the loss of current range and future range potential.

## Supporting Information

S1 Appendix Range Size Changes. Summary of projected changes in range size and losses of current range, by season. These are used to assign birds to Audubon climate sensitivity categories.
(XLSX)

S2 Appendix Projected Species Gains and Losses by State or Province. Projected changes in the number of species occupying in US states (except Hawaii) and provinces in Canada. There are 36 sets of projections for each place (2 seasons × 2 responses (gains/losses) × 3 time periods (2020, 2050, 2080) × 3 climate change scenarios (A2, A1B, B2). Values represent the mean change (gain or loss) within each State or Province.
(XLSX)

S3 Appendix Projected Species Gains and Losses by LCC. Projected changes in the number of species occupying all the landscape conservation cooperatives (LCCs) in the US and Canada (except Hawaii). There are 36 sets of projections for each LCC (2 seasons × 2 responses (gains/losses) × 3 time periods (2020, 2050, 2080) × 3 climate change scenarios (A2, A1B, B2). Values represent the mean change (gain or loss) within each LCC.
(XLSX)

S4 Appendix Projected Species Gains and Losses by BCR. Projected changes in the number of species occupying all the bird conservation regions (BCRs) in the US and Canada (except Hawaii). There are 36 sets of projections for each BCR (2 seasons × 2 responses (gains/losses) × 3 time periods (2020, 2050, 2080) × 3 climate change scenarios (A2, A1B, B2). Values represent the mean change (gain or loss) within each BCR.
(XLSX)

S5 Appendix Variance Components Analysis Results. Percentage of variation explained by covariates for the four main response variables considered in this study: relative change in range size and current range stability in both breeding and non-breeding seasons.
(XLSX)

S6 Appendix Migratory Status and Habitat Affiliations. Migratory status based on either [43] or assigned by authors. Habitat affiliations are from [39]. Note that a single species can have more than one habitat affiliation.
(XLSX)

S7 Appendix Climate Sensitivity and Conservation Status. Audubon climate sensitivity in relation to existing conservation status from IUCN, US government (Fed and BCC), Partners in Flight (PIF) and Audubon studies (WatchList and Common Birds in Decline).
(XLSX)

S8 Appendix BRT Variable Contributions. Variable contributions for covariates used in the boosted regression tree (BRT) analyses used to model species distributions. The values range between 0 and 100, and sum to 100 for each model (i.e., within a species/season). Larger values are indicative of covariates that have more influence.
(XLSX)

S9 Appendix Evaluation Information. Statistics associated with species sampling, model performance, and range comparisons.
(XLSX)

S10 Appendix All Data in S1–S9. All fields associated with species level data used in this climate sensitivity study.
(XLSX)

S1 Meta Data Descriptions of the data included in S1–S10 Appendices. (XLSX)
